# An efficient gene delivery method utilizing the regenerative capacity of alfalfa (*Medicago sativa*)

**DOI:** 10.1111/tpj.71195

**Published:** 2026-10-29

**Authors:** Jinghui Gao, Houyin Xia, Yajun Wu, Changgen Xie, Jiawen Peng, Yilin Cui, Yiyang Liu, Luna Tan, Chun Li, Qian Li, Jie An, Peizhi Yang

**Affiliations:** ^1^ College of Grassland Agriculture Northwest A&F University Yangling 712100 Shaanxi China; ^2^ Department of Biology and Microbiology South Dakota State University Brookings South Dakota 57007 USA; ^3^ State Key Laboratory for Crop Stress Resistance and High‐Efficiency Production, College of Life Sciences Northwest A&F University Yangling 712100 China; ^4^ College of Grassland Science Xinjiang Agricultural University Urumqi 830052 China

**Keywords:** stem regeneration, multiplex editing, tissue culture‐free, perennial plants

## Abstract

Alfalfa (*Medicago sativa*), an autotetraploid species, is a globally important forage crop. Traditional genetic transformation methods predominantly rely on leaf‐derived callus, a process that is complex, inefficient, and particularly challenging to generate stable transgenic lines in polyploid plants. To address these limitations, we developed an efficient transformation system that bypasses callus induction by directly exploiting stem regeneration. Our protocol enables the generation of stable gene‐edited alfalfa plants from shoot tips. It accommodates stem explants from various developmental stages and significantly reduces the time required. Moreover, this approach was effective across multiple alfalfa cultivars, and preliminary proof‐of‐concept experiments in *Nicotiana tabacum* and *Solanum lycopersicum* suggested potential transferability beyond alfalfa. This study represents a substantial advancement in perennial plant transformation and provides a robust and practical platform for optimizing stem regeneration‐based genetic modification systems.

## Significance Statement

This study converts the intrinsic regenerative capacity of alfalfa shoot tips and stem segments into a practical gene delivery and CRISPR/Cas9 editing system. By defining an early shoot‐tip developmental window that minimizes chimerism and validating multiple target genes, cultivars, and explant types, this method provides a rapid callus‐free platform for functional genomics and molecular breeding in polyploid perennial forage crops.

## INTRODUCTION

Alfalfa (*Medicago sativa*), known as the ‘Queen of Forage’, is one of the most important forage crops in the world, with approximately 45 million hectares cultivated globally as a primary livestock feed, and is the fourth largest field crop in the United States, after corn, soybeans, and wheat. As a recalcitrant, autotetraploid, perennial legume, alfalfa exhibits exceptional regenerative capabilities, allowing it to be routinely propagated by cutting and rooting of stem segments. However, existing alfalfa transformation systems rely heavily on lengthy and labor‐intensive tissue culture procedures (Chen et al., 2020, 2024; Fu et al., 2015; Samac & Austin‐Phillips, 2006). These limitations result in inconsistent transformation efficiencies, particularly for polyploid crops and perennial species, which are further constrained by low callus induction and regeneration rates (Li, Sun, et al., 2024). Developing more efficient and broadly applicable transformation methods has thus become a critical goal in alfalfa biotechnology.

In their natural environments, alfalfa, as a perennial species, exhibits remarkable regenerative capacities enabling the development of new shoots and roots from primordia, distinct from conventional somatic regeneration. Notably, perennial stem segments can regenerate without complex, time‐consuming tissue culture processes, making them ideal explants for genetic transformation (Cao et al., 2023, 2024, 2026; Zhao & Wang, 2024). However, gene delivery methods that leverage the regenerative potential of alfalfa remain unexplored. We hypothesized that the regenerative potential of perennials could be directly harnessed for genetic transformation. Here, we present a more efficient genetic transformation system that leverages the regenerative capacity of alfalfa stems, involving both gene overexpression and CRISPR/Cas9‐mediated gene editing. We evaluated the applicability and stability of this system across different alfalfa genotypes to explore its potential for transforming other plant species. Specifically, this system could successfully enable the targeted editing of multiple genes in various alfalfa cultivars.

## RESULTS

### Efficient genetic transformation of alfalfa via shoot‐tip regeneration

To develop a transformation system that directly exploits the regenerative potential of alfalfa, we used shoot‐tip explants excised from young seedlings before true leaf emergence. The upper 3–5 mm region of 3–5‐day‐old seedlings was collected after removing cotyledons and roots, leaving a short hypocotyl‐associated shoot‐tip segment that retained the apical meristem and young shoot primordium (Figure S1). This explant type was selected because it retains the apical meristem and young shoot primordium and can regenerate shoots and roots without callus induction. RT‐qPCR analysis further showed that shoot‐tip tissues exhibited elevated expression of multiple meristem‐ and cell division‐associated genes, supporting their high regenerative competence (Figure 1a; Figure S2). Shoot tips infected with an *EGFP* reporter construct produced *EGFP*‐positive shoots (Figure 1b,c). PCR analysis confirmed the presence of the transgene in regenerated shoots (Figure S3). Southern blot analysis using a DIG‐labeled *hyg*‐specific probe detected hybridization signals in representative transgenic lines but not in the wild‐type control, confirming stable genomic integration of the introduced T‐DNA (Figure S4). *RUBY*‐positive explants and regenerated plants displayed visible red‐purple pigmentation in newly developed shoots, leaves and roots, whereas uninfected controls did not (Figure 1d; Figure S5) (He et al., 2020; Yi et al., 2024). These results show that the shoot‐tip transformation route supports visual identification and recovery of stable transgenic alfalfa plants within approximately 2 months.

**Figure 1:**
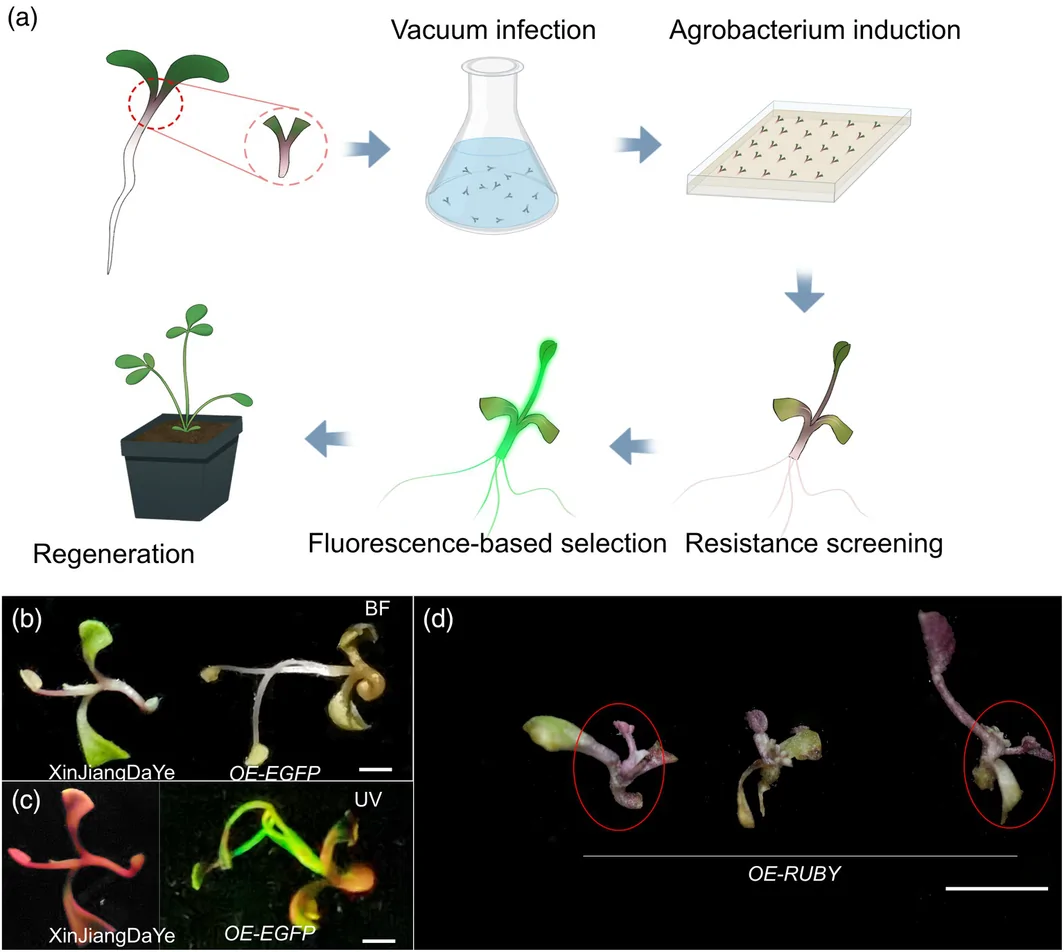
Establishment of a shoot‐tip transformation system for transgene expression in alfalfa. (a) Schematic workflow of the alfalfa shoot‐tip transformation system. Shoot‐tip explants containing the apical meristem and a short hypocotyl segment were excised from young seedlings, subjected to *Agrobacterium tumefaciens*‐mediated vacuum infiltration, co‐cultivated on induction medium, screened under antibiotic selection, and identified by fluorescence‐based selection before regeneration into complete plants. (b, c) Representative bright‐field (BF; b) and ultraviolet excitation (UV; c) images of uninfected XinJiangDaYe shoot tips and *OE‐EGFP* transformed shoot tips. Strong EGFP fluorescence was detected in transformed tissues under UV excitation. (d) Representative *OE‐RUBY* transformed shoot‐tip explants showing visible red‐purple pigmentation in newly regenerated tissues. Red circles indicate RUBY‐positive regions. Scale bars: 1 cm.

### Shoot‐tip transformation system for gene editing in alfalfa

Building on these findings, we evaluated whether the shoot‐tip system supported genome editing. We targeted the *M. sativa* *PHYTOENE DESATURASE* gene (*MsPDS*), for which disruptive editing produces an albino or pale phenotype (Corona et al., 1996). Regenerated *MsPDS*‐edited Tourist plants displayed albino or pale phenotypes relative to the non‐edited control (Figure 2a; Figure S6). Across three independent experiments, regeneration rates were 96.0%, 88.0%, and 95.0%, and editing efficiencies were 43.8, 33.0, and 22.1%, respectively (Figure 2b,c; Table S1). In total, 92 edited plants were recovered from 279 regenerated plants, including 17 homozygous/tetra‐allelic edited plants (6.1% of regenerated plants). Sequence analysis of representative line *#Mspds‐5* identified substitutions, frameshift deletions, and an in‐frame deletion at the target region (Figure 2d; Figure S6). Chimerism was assessed from target‐locus genotyping patterns rather than reporter fluorescence. No chimeric genotype was detected among the 92 *MsPDS*‐edited plants generated from 3‐ to 5‐day‐old shoot tips; by contrast, 15 of 17 edited plants generated from 7‐day‐old shoot tips were chimeric (88.2% of edited plants; 11.5% of regenerated plants), and editing efficiency was lower (Tables S1 and S2). We therefore used 3–5‐day‐old seedlings for all subsequent shoot‐tip editing experiments.

**Figure 2:**
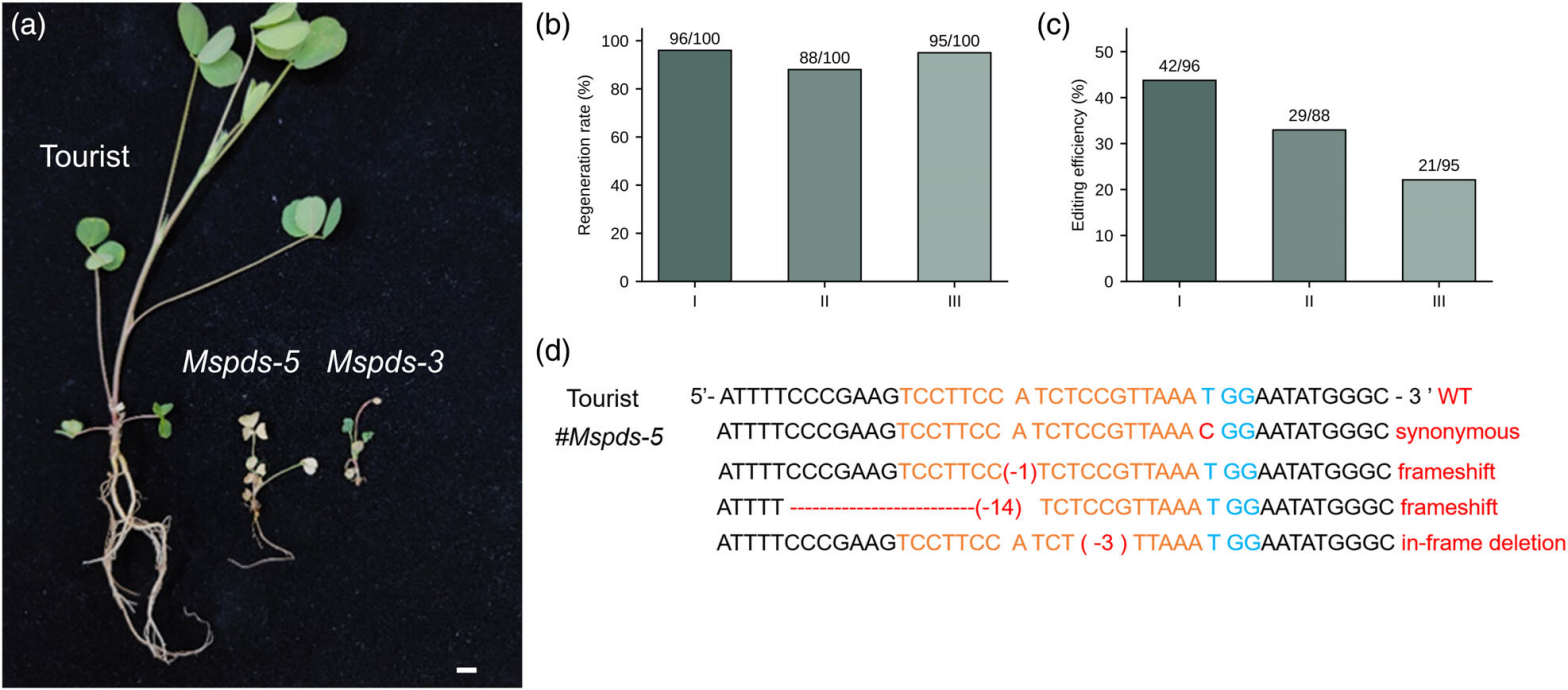
Generation and validation of *MsPDS*‐edited alfalfa plants using the shoot‐tip transformation system. (a) Representative phenotype of *MsPDS*‐edited plants generated from Tourist shoot‐tip explants. The edited lines #*Mspds‐5* and #*Mspds‐3* displayed albino or pale phenotypes compared with the non‐edited Tourist control, consistent with disruption of carotenoid biosynthesis. (b) Regeneration rates in three independent experiments using 3–5‐day‐old shoot‐tip explants. Regeneration rate was calculated as regenerated plants divided by total explants × 100%. (c) *MsPDS* editing efficiencies in the same experiments. Editing efficiency was calculated as edited plants divided by regenerated plants × 100%. (d) Sequence alignment of representative edited alleles from #*Mspds‐5*. Compared with the Tourist wild‐type sequence, #*Mspds‐5* carried multiple mutation types at the target region, including nucleotide substitution, frameshift deletions, and an in‐frame deletion. Orange sequences indicate the sgRNA target region, blue bases indicate PAM or key target‐site nucleotides, red dashed lines and brackets indicate deleted nucleotides and deletion sizes, and red text denotes the predicted mutation consequence. Scale bar: 1 cm.

### The shoot‐tip system supports functional editing in multiple alfalfa cultivars

To evaluate functional editing, we targeted *MsSIG6*, *MsDG1*, and *MsBRI1* in the alfalfa cultivar XinJiangDaYe using dual‐sgRNA CRISPR/Cas9 constructs (Figure S7) (Chi et al., 2008, 2010; Wang et al., 2001; Zhou et al., 2004). *MsSIG6*‐edited plants showed chlorosis, *MsDG1*‐edited plants showed early leaf yellowing and dwarfing, and *MsBRI1*‐edited plants displayed shortened petioles and impaired leaf development (Figure 3a–c). Homozygous/tetra‐allelic edited lines were recovered for all three targets. Sequence analysis of representative lines *#Mssig6‐10*, *#Msdg1‐13*, and *#Msbri1‐32* confirmed multiple deletions and frameshift mutations at the target regions (Figure S8).

**Figure 3:**
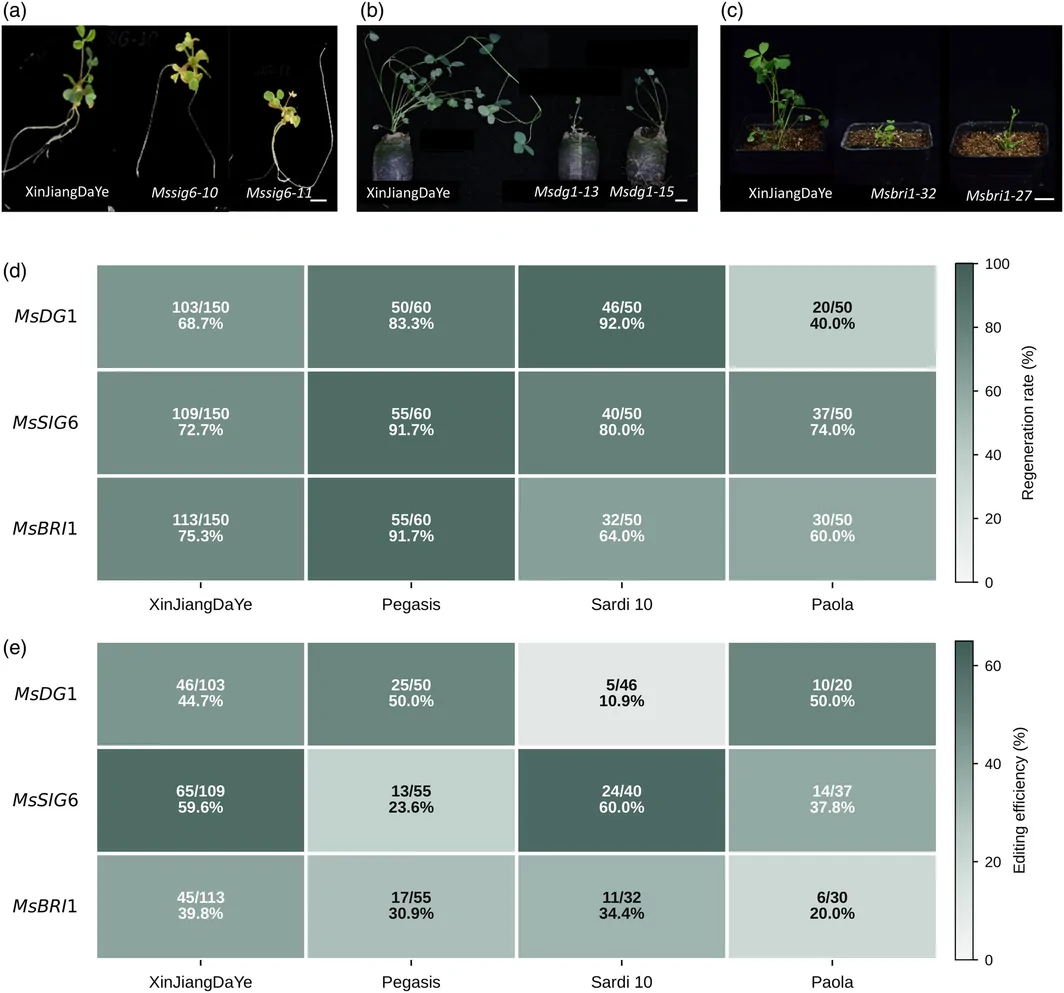
Functional gene editing and genotype applicability of the alfalfa shoot‐tip transformation system. (a–c) Representative phenotypes of *MsSIG6‐*, *MsDG1‐*, and *MsBRI1‐edited* plants in the XinJiangDaYe background. *MsSIG6*‐edited plants showed chlorotic phenotypes (a), *MsDG1*‐edited plants showed growth inhibition and dwarfing phenotypes (b), and *MsBRI1*‐edited plants displayed developmental defects, including reduced plant height and impaired leaf development. (d) Heatmap of regeneration rates for *MsDG1*, *MsSIG6*, and *MsBRI1* across XinJiangDaYe, Pegasis, Sardi 10 and Paola. Regeneration rate was calculated as regenerated plants divided by total explants × 100%. (e) Heatmap of editing efficiencies for the same gene–cultivar combinations. Editing efficiency was calculated as edited plants divided by regenerated plants × 100%. Scale bars: 1 cm.

To evaluate applicability across alfalfa genetic backgrounds, we tested editing of *MsDG1*, *MsSIG6*, and *MsBRI1* in four cultivars. Regeneration and editing efficiencies varied by target and cultivar (Figure 3d,e; Table S3). Significant cultivar‐dependent differences in editing efficiency were detected for *MsDG1* and *MsSIG6* (adjusted *P* < 0.001), whereas the difference for *MsBRI1* was not significant (*P* = 0.210). No chimeric genotype was detected among the 281 edited plants generated in these experiments. Representative edited phenotypes were observed across Pegasis, Sardi 10, and Paola (Figures S9–S11). These results demonstrate applicability across the tested genetic backgrounds while also revealing target‐ and cultivar‐dependent variation.

To test proof‐of‐concept transferability beyond alfalfa, we applied the shoot‐tip procedure to young *Nicotiana tabacum* and *Solanum lycopersicum* seedlings. *EGFP*‐positive tissues were recovered in both species under ultraviolet excitation. Positive frequencies, calculated as *EGFP*‐positive plants divided by total explants, were 10.0% in *N. tabacum* and 6.3% in *S. lycopersicum* (Figure S12; Table S4). These preliminary experiments suggest potential transferability but do not establish broad cross‐species applicability.

### Stem‐segment transformation system for gene editing in alfalfa

Because mature alfalfa plants can be vegetatively propagated from stem cuttings, we tested a complementary transformation route using stem segments containing an axillary bud. Segments were excised from 40‐day‐old WL363 plants, infected with *A. tumefaciens* by vacuum infiltration, co‐cultivated, and regenerated by cutting propagation (Figure 4a; Figure S13). *EGFP* fluorescence was detected in emerging shoots (Figure 4b,c). Southern blot analysis using the same DIG‐labeled HYG‐specific probe detected hybridization signals in representative stem‐segment‐derived transgenic lines but not in the wild‐type control (Figure S4). *EGFP* was detected in 38/38 primary branches (100%), 20/22 secondary branches (90.9%), and 10/14 tertiary branches (71.4%) during successive vegetative propagation (Table S5). HYG‐specific genomic PCR detected the expected 245‐bp fragment in all analyzed branches (Table S5), including branches with weak or undetectable *EGFP* fluorescence. The decline in detectable *EGFP* fluorescence is therefore unlikely to result primarily from widespread T‐DNA loss and may instead reflect heterogeneity or reduction in reporter expression during propagation. Transformation with an *MsSGR* overexpression construct was successful in WL363, Pegasis and Paola. PCR and expression analyses confirmed the presence and expression of the introduced *MsSGR* transgene (Figure S14). After dark treatment, OE‐*MsSGR* plants showed stronger yellowing than WL363 controls (Figure 4d), supporting successful transgene expression (Li et al., 2026; Zhou et al., 2011). Transgene‐positive plant frequencies were 33.3, 47.2, and 44.4%, respectively, with no significant difference among cultivars (Pearson's χ^2^ test, *P* = 0.547; Table S6).

**Figure 4:**
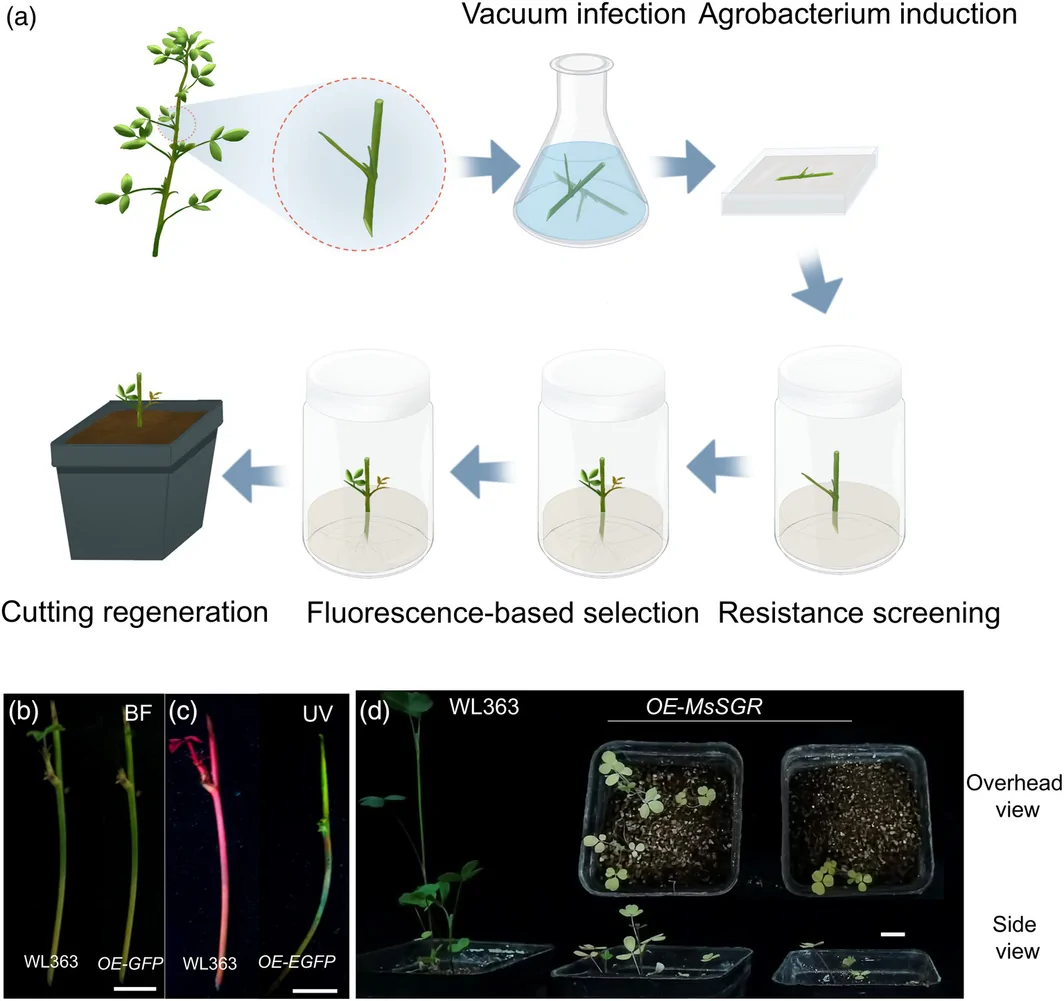
Transgene expression and phenotypic validation using the mature stem‐segment transformation system. (a) Workflow of the mature stem‐segment transformation system. Stem segments containing an axillary bud were excised from mature alfalfa plants, vacuum infiltrated with *Agrobacterium tumefaciens*, co‐cultivated, selected, and regenerated by cutting propagation. (b, c) Representative bright‐field (BF; b) and ultraviolet excitation (UV; c) images of WL363 control and OE‐*EGFP* stem segments. (d) Representative phenotypes of WL363 control and OE‐*MsSGR* plants after 3 days of dark treatment. OE‐*MsSGR* plants showed enhanced yellowing relative to WL363. Scale bars: 1 cm.

Finally, we tested whether mature stem segments supported genome editing. A CRISPR/Cas9 construct targeting *MsDG1* was introduced into WL363 stem segments. After regeneration and cutting propagation, 16 edited plants were recovered from 60 explants, including one homozygous/tetra‐allelic edited plant and 15 heterozygous or partially edited plants (Table S7). Representative line #*Msdg1‐1* showed a dwarf phenotype and carried multiple edited alleles at the *MsDG1* target site (Figure S15). These findings show that mature stem segments provide a complementary route for both transgene expression and genome editing.

## DISCUSSION

Conventional plant transformation commonly requires callus induction and regeneration (Brandizzi et al., 2025). By contrast, alfalfa shoot tips and axillary‐bud‐containing stem segments can regenerate directly from pre‐existing meristems. Shoot apical and axillary meristems sustain continued organogenesis, although the molecular state of each explant type is not identical (Lee et al., 2011; Liu et al., 2024; Ogura et al., 2023; Zhai & Xu, 2021). In our RT‐qPCR analysis, young shoot tips showed enrichment of the cell‐cycle marker *CYCB1;1* and regeneration‐associated *PLT5*, together with elevated *WOX5* and *WUS* expression. Callus showed the highest *WOX5* and *WUS* expression, whereas stem segments displayed a more restricted regeneration‐associated profile. These data support the developmental competence of shoot tips but do not establish the identity of the cells initially transformed by *Agrobacterium*. Direct confirmation will require time‐resolved histology, meristem‐specific reporters, or lineage tracing.

The variation in editing efficiency among *MsSIG6*, *MsDG1*, and *MsBRI1* is consistent with the target dependence of CRISPR/Cas9 activity. sgRNAs can differ substantially in activity because of sequence composition, guide‐RNA structure, and target‐site properties (Zhu et al., 2020). In autotetraploid alfalfa, Zhao et al. (2024) reported editing frequencies ranging from undetectable levels to 83.0% among sgRNAs targeting PALM1. Chromatin context can also influence Cas9 accessibility; in *Arabidopsis*, DNA methylation and chromatin features were associated with differences in mutagenesis efficiency of up to approximately 250‐fold (Weiss et al., 2022). Allelic sequence variation adds another layer of target dependence in autotetraploid alfalfa, where complete mutants require editing of multiple homologous alleles. Finally, target‐specific effects on regeneration or survival may influence the proportion of edited plants recovered. The present study does not separate these contributions; sgRNA pre‐screening and characterization of target‐locus sequence and chromatin context should improve editing consistency.

By exploiting this regenerative capacity, the present system avoids callus induction and shortens the time required to recover edited plants. Shoot tips are suitable for rapid generation of T0 mutants, whereas stem segments provide a complementary route for vegetative propagation, complementation and construction of additional edited materials. Cultivated alfalfa is a predominantly outcrossing, self‐incompatible autotetraploid with tetrasomic inheritance (Li, Liu, et al., 2024; Pereira & Erickson, 1995; Santoro et al., 2025). Sexual transmission therefore cannot be evaluated in the same manner as in self‐compatible diploid species, and transgene‐negative progeny can arise through normal segregation rather than instability of the original integration event. In this study, Southern blotting and successive‐branch genomic PCR were used to evaluate stable integration and retention during vegetative propagation. Controlled crosses with sufficiently large progeny populations will be required to characterize sexual transmission and segregation.

Despite its applicability across the alfalfa genetic backgrounds tested here, the system retains genotype dependence. Regeneration and editing efficiencies varied among cultivars. Genotype‐dependent regeneration is a recognized limitation in alfalfa transformation, and marked differences in regenerative capacity can occur under similar culture conditions (Zhang et al., 2010). Genotype dependence also remains a major constraint in plant engineering more broadly (Youngstrom et al., 2024). The observed variation may reflect intrinsic regenerative competence, susceptibility to *Agrobacterium*‐mediated delivery, sgRNA activity, target‐locus chromatin context, allelic sequence variation, or a combination of these factors. Because infection efficiency and regenerative competence were not independently quantified in each genotype, their relative contributions cannot be distinguished. Broader germplasm testing and systematic comparison of these components will be needed to improve consistency.

## PLANT MATERIALS AND METHODS

### Vector construction

All PCR cloning was performed using 2× Rapid Taq Master Mix (Vazyme, Nanjing). We used the pEarleyGate 100 vector backbone to construct the *enhanced green fluorescent protein* (*EGFP*), *M. sativa Stay‐Green* (*MsSGR*)‐*EGFP*, or *RUBY* gene constructs. For gene editing vectors, we utilized PHEE401, provided by Dr. Qijun Chen's laboratory, with pCambia as the backbone (Xing et al., 2014). The *Medicago truncatula* U6 promoter drove the single guide RNA (sgRNA), while the Cas9 protein was driven by the 35S promoter. PCR products were recovered from gels, and vector digestions were purified using PCR purification kits for subsequent *in vitro* or *in vivo* recombination reactions. Approximately 5–10 μl of ligation products were transformed into competent *Escherichia coli* cells. Transformants were plated on kanamycin‐selective plates and incubated at 37°C for 12 h, followed by colony PCR screening. Ten colonies were selected for both 1.5 ml culture and PCR identification. sgRNA sequences are listed in Table S8.

### Plant materials and growth conditions

Alfalfa (*M. sativa* L.) cultivars Tourist, XinJiangDaYe, Pegasis, Sardi 10, Paola and WL363 were used. Tourist was used to establish the shoot‐tip system and for *MsPDS* editing. XinJiangDaYe was used for functional validation of *MsSIG6*, *MsDG1*, and *MsBRI1*, whereas Pegasis, Sardi 10 and Paola were used to assess applicability across genetic backgrounds. WL363 was used to establish the mature stem‐segment system. *N. tabacum* and *S. lycopersicum* seedlings were used only for preliminary proof‐of‐concept assessment of transferability beyond alfalfa.

For shoot‐tip transformation, surface‐sterilized seeds were germinated on seed germination medium under a 12‐h light/12‐h dark photoperiod at 25 ± 2°C and a light intensity of 2000 lx. Shoot tips were excised from 3‐ to 5‐day‐old seedlings before true leaf emergence. For mature stem‐segment transformation, 40‐day‐old WL363 plants were used, and segments from the third to fifth nodes were collected after leaf removal while retaining one axillary bud per segment.

Regeneration rate was calculated as regenerated plants divided by total explants × 100%, whereas editing efficiency was calculated as edited plants divided by regenerated plants × 100%. For mature stem‐segment transformation, transgene‐positive plant frequency was calculated as PCR‐confirmed positive plants divided by regenerated plants × 100%. For the preliminary tobacco and tomato experiments, positive frequency was calculated as *EGFP*‐positive plants divided by total explants × 100%. The denominator for each proportion is also specified in the relevant figure or table legend.

### *Agrobacterium* preparation for plant transformation

*Agrobacterium tumefaciens* strain EHA105 was employed to deliver both transgene and gene editing constructs. For plant transformation, a single and confirmed *Agrobacterium* EHA105 colony harboring the editing construct was cultured in 50 ml of LB liquid medium (with 50 mg L^−1^ rifampicin and 50 mg L^−1^ kanamycin) at 28°C, 220 rpm for 18 h. The culture was then transferred to 300 ml of LB medium (containing 50 mg L^−1^ rifampicin and 50 mg L^−1^ kanamycin) and further grown at 28°C, 220 rpm for 6–10 h. When the optical density at 600 nm (OD_600_) reached 0.4–0.6, bacterial cells were collected by centrifugation at room temperature (4000 rpm, 10 min) and resuspended in MS liquid medium for plant transformation. Culture‐medium compositions are listed in Table S9.

### Shoot tip transformation system

Alfalfa seeds were disinfected by immersion in 30% sodium hypochlorite (NaClO) for 18 min, followed by 3–5 washes with sterile water. Sterilized seeds were dried on sterile filter paper, placed onto seed germination medium, and incubated in a growth chamber (25 ± 2°C, 12‐h light/12‐h dark photoperiod, light intensity of 2000 lx) for 3–4 days. 3 to 5‐day old seedlings before true leaf emerges (cotyledons developed but not fully expanded) were selected. The shoot tips portion (3–5 mm including the apical meristem without cotyledons) were excised, immersed in the bacterial suspension described above, and subjected to vacuum infiltration for 20–30 min until they sank. The shoot tips were incubated in the bacterial solution without vacuum for another 30 min. The tips were then removed from the bacterial suspension and blotted dry with sterile filter paper before being transferred to co‐cultivation medium for 3 days in a dark incubator at 25 ± 2°C. Subsequently, shoot tips were transferred to the selection medium containing 0.5 g L^−1^ timentin to wash away bacterial clumps through gentle mixing. The shoot tips were then rinsed three times with sterile water. After blotting dry with sterile filter paper, the tips were placed back in selection agar medium. Approximately 2 weeks later, surviving alfalfa plants with true leaves were assessed for fluorescent protein expression. Positive plants were transferred to vermiculite for rooting and further growth. For transformation in *N*. *tabacum* and *S*. *lycopersicum*, shoot tips of 3–4‐day‐old seedlings were used.

### Stem segment transformation system

Young alfalfa stem segments (~5 cm long) with an axillary bud or terminal buds were disinfected by immersion in 30% NaClO for 20 min, followed by 3–5 washes with sterile water. Sterilized stem segments were blotted dry with sterile filter paper and then immersed in the *Agrobacterium* suspension, as previously described. The segments were subjected to vacuum infiltration for 30–40 min until they sank, followed by incubation without vacuum for another 30 min. The bacterial suspension and explants were then separated, and the explants were blotted dry with sterile filter paper before being transferred to co‐cultivation medium in a dark incubator at 25°C for 3 days. After co‐cultivation, stem segments were transferred to bottles containing selection medium and gently shaken with 0.5 g L^−1^ timentin to remove bacterial clumps. Segments were then washed three times with sterile water, dried on sterile filter paper, and placed back in selection agar medium. Fluorescent protein expression was observed in emerging young shoots, which were marked, subjected to cutting propagation, and transferred to vermiculite for rooting and normal growth.

### Fluorescence observation

*EGFP* fluorescence was initially screened using a LUYOR‐3415RG handheld dual‐fluorescent protein observation lamp. Putative *EGFP*‐positive tissues, including transformed shoot tips, regenerated shoots, roots, and stem segments, were observed under the handheld fluorescence lamp, and the corresponding bright‐field images were recorded for comparison. Non‐transformed WT tissues were used as negative controls in each observation.

### Southern blot analysis

Genomic DNA was extracted from wild‐type and representative transgenic alfalfa plants. Approximately 10 μg of genomic DNA was completely digested with EcoRI and separated on a 0.7% agarose gel by electrophoresis at 25 V overnight. The DNA was transferred onto a positively charged HyBond N+ nylon membrane by upward capillary transfer and immobilized at 80°C for 2 h.

A 245‐bp fragment of the *hyg*romycin‐resistance selectable marker gene (*hyg*) was amplified using HYG‐SB‐F (5′‐CCAAGCTCTGATAGAGTTGGTC‐3′) and HYG‐SB‐R (5′‐GGATTTCGGCTCCAACAATGTC‐3′). The PCR product was labeled with DIG using the PCR DIG Probe Synthesis Kit (Roche). Following prehybridization, membranes were hybridized overnight with the denatured DIG‐labeled probe. After stringent washing, hybridization signals were detected with anti‐DIG alkaline phosphatase and CSPD chemiluminescent substrate according to the manufacturer's instructions.

### Dark treatment of *OE‐MsSGR* plants

PCR‐confirmed *OE‐MsSGR‐EGFP* transgenic plants and WL363 control plants at the same developmental stage were used for dark‐treatment assays. *Plants* were transferred to complete darkness at 25°C for 3 days. After dark treatment, plant phenotypes were photographed, and leaves or young shoots were collected for RNA extraction and RT‐qPCR analysis. WT plants maintained under the same dark‐treatment conditions were used as controls. The yellowing phenotype of *OE‐MsSGR* plants was evaluated after dark treatment.

### DNA extraction and sequencing

Genomic DNA was extracted from leaves using the sodium dodecyl sulfate method. Target‐locus primers were designed around each sgRNA site, and PCR amplicons were analyzed by Sanger sequencing (Wuhan Boyuan Biotechnology Co., Ltd.). Primer sequences for transgene validation, HYG‐specific genomic PCR, and target‐site sequencing are listed in Table S10.

### Expression analysis by RT‐qPCR

To evaluate the meristematic characteristics of different alfalfa tissues, shoot tips from 3 to 5‐day‐old seedlings, stem segments, callus, and fully developed leaves were collected for RNA extraction. Total RNA was isolated with TRIzol reagent, and first‐strand cDNA was synthesized using a reverse‐transcription kit. Expression of *BBM*, *CYCB1;1*, *PLT5*, *WOX5*, and *WUS* was measured by RT‐qPCR. *EF‐1α* was used as the internal reference gene, and leaf tissue was used as the calibrator. Relative transcript abundance was calculated by the 2^−ΔΔCt^ method. Data are presented as mean ± SEM from three independent biological replicates. Statistical analyses were performed on ΔCt values by one‐way ANOVA followed by Tukey's HSD test. Different lowercase letters indicate significant differences among tissues at *P* < 0.05. Primer sequences are listed in Table S10.

For *MsSGR* expression analysis, total RNA was extracted with TRIzol reagent and reverse transcribed. RT‐qPCR was performed with SYBR Green Master Mix, and relative expression was calculated by the 2^−ΔΔCt^ method using *MsActin* as the internal reference. Data are presented as mean ± SE. Primer sequences are listed in Table S10.

### Statistical analysis

Statistical analyses were performed using SPSS Statistics version 25.0 and Python‐based statistical packages where appropriate. RT‐qPCR comparisons were conducted on ΔCt values. Differences among tissue types were evaluated by one‐way ANOVA followed by Tukey's HSD test; two‐group comparisons used two‐tailed Student's *t*‐tests where appropriate. Proportion‐based transformation and editing data were compared among cultivars with Pearson's chi‐squared tests followed, where appropriate, by pairwise Fisher's exact tests with Holm adjustment. Adjusted *P* < 0.05 was considered statistically significant.

## AUTHOR CONTRIBUTIONS

PY, JG, YW, and CX designed and supervised this research. HX, JP, YC, YL, LT, CL, and QL conducted experiments. HX, JP, YC, and JA planned the experiments. HX and JP performed the data analysis. HX wrote the paper. All authors reviewed the paper draft. The authors wish it to be known that, in their opinion, Jinghui Gao, Houyin Xia, Yajun Wu, and Changgen Xie contributed equally to this work and share first authorship. 

## CONFLICT OF INTEREST

The authors declare no competing interests.

## Supporting information


**Figure S1.** Alfalfa shoot tip transformation system. (a) 3–5‐day‐old sterile alfalfa seedling used for shoot‐tip explant preparation. The red circle indicates the cutting region around the cotyledonary node and upper hypocotyl, where the shoot tip was excised. (b) Excised shoot‐tip explant containing the young shoot apex and a short hypocotyl segment for subsequent *Agrobacterium*‐mediated infection. (c) Shoot‐tip explants placed on co‐cultivation medium after *Agrobacterium* infection to facilitate bacterial attachment and initiate the transformation process. Multiple infected explants are shown arranged on the medium. Scale bars: 1 cm.
**Figure S2.** Expression of meristem‐ and cell division‐associated genes in different alfalfa tissues. Relative expression levels of *BBM* (a), *CYCB1;1* (b), *PLT5* (c), *WOX5* (d) and *WUS* (e) in leaves, stem segments, shoot tips and callus were determined by RT‐qPCR. *EF‐1α* was used as the internal reference gene, and leaf tissue was used as the calibrator for calculation of relative expression using the 2^−ΔΔCt^ method. Data are presented as mean ± SEM of three independent biological replicates. Statistical comparisons were performed using ΔCt values by one‐way ANOVA followed by Tukey's HSD multiple‐comparison test. Different lowercase letters indicate significant differences among tissues at *P* < 0.05.
**Figure S3.** Construction of the EGFP overexpression vector and identification of *EGFP*‐positive transgenic alfalfa plants. (a) Schematic representation of the *EGFP* transgenic vector used for alfalfa shoot‐tip transformation. The T‐DNA region is flanked by the left border (LB) and right border (RB) and contains the 2×35S promoter, TMV translational enhancer, EGFP reporter gene, OCS terminator, and the *hygromycin resistance* gene (*hyg*). (b and c) Representative phenotypes of untransformed XinJiangDaYe WT seedlings and *OE‐EGFP* transgenic alfalfa seedlings. Under bright‐field imaging (BF), the overall morphology of XinJiangDaYe WT and *OE‐EGFP* seedlings is shown. Under ultraviolet excitation (UV), strong green fluorescence was detected in the roots and shoot tissues of *OE‐EGFP* plants. (d) PCR‐based validation of transgene‐positive regenerated shoots. *OE‐EGFP* samples produced the expected EGFP‐specific amplification band, consistent with the plasmid positive control, while no corresponding band was detected in the negative control. Scale bars, 1 cm.
**Figure S4.** Southern blot analysis of shoot‐tip‐ and stem‐segment‐derived transgenic alfalfa lines. Genomic DNA from wild‐type and representative transgenic alfalfa plants generated using the shoot‐tip and mature stem‐segment transformation systems was digested with EcoRI and hybridized with a DIG‐labelled probe specific to the hygromycin‐resistance selectable marker gene (hyg). The shoot‐tip‐derived and stem‐segment‐derived samples were analysed on the same Southern blot membrane and are presented together in this figure. Specific hybridization signals were detected in representative transgenic lines generated by both transformation routes, whereas no corresponding signal was detected in the wild‐type control. M, DNA Molecular‐Weight Marker II; WT, wild‐type control.
**Figure S5.** Visual application of the *RUBY* reporter gene in the alfalfa shoot‐tip transformation system. (a) Schematic representation of the *RUBY* expression vector used for alfalfa shoot‐tip transformation. The T‐DNA region is flanked by the left border (LB) and right border (RB) and contains the 2×35S promoter, TMV translational enhancer, *RUBY* reporter gene, OCS terminator, and the *hygromycin resistance* gene (*hyg*). (b) Representative phenotypes of uninfected XinJiangDaYe WT plants and *OE‐RUBY* transgenic alfalfa plants. No visible red‐purple pigmentation was observed in XinJiangDaYe WT plants, whereas *OE‐RUBY* plants displayed clear red‐purple coloration in shoots, leaves, and roots. (c) PCR validation of *RUBY* regenerated buds. All eight *OE‐RUBY* regenerated buds produced the expected target band, consistent with the plasmid positive control, whereas no corresponding amplification was detected in the negative control. Scale bars, 1 cm.
**Figure S6.** Construction of the *MsPDS* CRISPR/Cas9 editing vector and sequence validation of representative edited plants. (a) Schematic representation of the CRISPR/Cas9 editing vector and sgRNA designed for *MsPDS*. The T‐DNA region contains the zCas9 expression cassette and the sgRNA expression cassette. zCas9 is driven by the 2×35S promoter and terminated by the rbcS E9t terminator, while *hyg* is used as the hygromycin‐resistance selectable marker gene. The sgRNA cassette consists of the U6‐26p promoter, the *MsPDS*‐targeting sgRNA sequence, and U6‐26t. The target sgRNA sequence for *MsPDS* is shown below the vector diagram. (b) Sanger sequencing chromatograms of different edited *MsPDS* alleles. Red boxes indicate mutation sites, showing representative base substitutions or deletion events at the target region.
**Figure S7.** Construction of CRISPR/Cas9 editing vectors for *MsSIG6*, *MsDG1*, and *MsBRI1*. (a–c) Schematic representations of the dual‐sgRNA CRISPR/Cas9 editing vectors targeting *MsSIG6* (a), *MsDG1* (b), and *MsBRI1* (c). Each T‐DNA region contains a zCas9 expression cassette driven by the 2×35S promoter and terminated by the rbcS E9t terminator, two sgRNA expression cassettes driven by the U6‐26p and U6‐29p promoters, and the hygromycin‐resistance marker gene *hyg*. The target sequences of sgRNA1 and sgRNA2 for each gene are shown below the corresponding vector diagram.
**Figure S8.** Validation of representative mutant alleles in *MsSIG6 ‐* , *MsDG1 ‐* , and *MsBRI1 ‐* edited plants. (a–c) Sequence alignments of representative edited alleles from *MsSIG6*‐edited line #*Mssig6‐10* (a), *MsDG1*‐edited line #*Msdg1‐13* (b), and *MsBRI1*‐edited line #*Msbri1‐32* (c). Compared with the XinJiangDaYe WT sequence, all three edited lines carried multiple mutation types at the target regions. Red dashed lines and brackets indicate deletions and deletion sizes, orange sequences indicate sgRNA target regions, blue bases indicate PAM or key target‐site nucleotides, and red text denotes the predicted mutation consequences.
**Figure S9.** Phenotypic characterization of gene‐edited plants in the Pegasis background. Representative phenotypes of Pegasis WT and *MsSIG6*‐, *MsDG1*‐ and *MsBRI1*‐edited plants generated using the shoot‐tip transformation system. Pegasis WT, *sig6‐39*, *dg1‐23* and *bri1‐32* are shown from left to right. The same cultivar‐matched WT plants served as the common control for the three edited genotypes because these materials were phenotypically evaluated within the same experimental batch. Scale bar, 1 cm.
**Figure S10.** Phenotypic characterization of gene‐edited plants in the Sardi 10 background. Representative phenotypes of Sardi 10 WT and *MsSIG6*‐, *MsDG1*‐ and *MsBRI1*‐edited plants generated using the shoot‐tip transformation system. Sardi 10 WT, *sig6‐9*, *dg1‐26* and *bri1‐35* are shown from left to right. The same cultivar‐matched WT plants served as the common control for the three edited genotypes because these materials were phenotypically evaluated within the same experimental batch. Scale bar, 1 cm.
**Figure S11.** Phenotypic characterization of gene‐edited plants in the Paola background. Representative phenotypes of Paola WT and *MsSIG6*‐, *MsDG1*‐ and *MsBRI1*‐edited plants generated using the shoot‐tip transformation system. Paola WT, *sig6‐11*, *dg1‐25* and *bri1‐25* are shown from left to right. The same cultivar‐matched WT plants served as the common control for the three edited genotypes because these materials were phenotypically evaluated within the same experimental batch. Scale bar, 1 cm.
**Figure S12.** Transformation validation of *EGFP* in *Nicotiana tabacum* and *Solanum lycopersicum*. (a) Green fluorescence signal of *Nicotiana tabacum OE*‐*EGFP t*ransgenic material under UV excitation. (b) Green fluorescence signal of *Solanum lycopersicum OE‐EGFP* transgenic material under UV excitation. Scale bars, 1 cm.
**Figure S13.** Alfalfa stem segment transformation system. (a) Mature alfalfa donor plant used for preparing stem‐segment explants. The red circle indicates the stem region selected for excision, mainly consisting of healthy green stem tissues. (b) Excised stem‐segment explant. Stem tissues were trimmed to an appropriate length for subsequent *Agrobacterium*‐mediated transformation. (c) Bright‐field (BF) image showing the overall morphology of the infected stem segment and root formation. Scale bars, 1 cm.
**Figure S14.** Construction of the *MsSGR‐EGFP* overexpression vector and molecular validation of transgenic alfalfa lines. (a) Schematic representation of the *MsSGR‐EGFP* transgenic expression vector. The T‐DNA region is flanked by the left border (LB) and right border (RB) and contains the 2×35S promoter, TMV translational enhancer, *MsSGR* coding sequence, *EGFP* reporter gene, OCS terminator, and the hygromycin‐resistance selectable marker gene *hyg*, enabling the generation of *MsSGR‐EGFP* overexpression materials. (b) PCR validation of *MsSGR‐EGFP* transgene‐positive buds. Target amplification bands were detected in OE‐*MsSGR‐EGFP* samples, whereas no corresponding band was observed in the negative control, confirming the presence of the transgene in regenerated buds. The DNA marker is shown on the left, with 500‐bp and 750‐bp bands indicated. (c) Relative expression levels of *MsSGR* in transgenic alfalfa plants. WT was used as the reference control. **P < 0.01, ***P < 0.001 considered statistically significant.
**Figure S15.** Phenotype and target‐site sequence validation of the *MsDG1*‐edited plant #*Msdg1*‐1 generated using the stem‐segment transformation system. (a) Representative phenotype of the WL363 control plant and the *MsDG1*‐edited plant #*Msdg1*‐1. (b) Sequence alignment of different edited alleles at the *MsDG1* target site in #*Msdg1*‐*1*, using the WL363 wild‐type sequence as the reference. Multiple allele types were detected in #*Msdg1*‐*1*. Orange sequences indicate the sgRNA target region, blue bases indicate the PAM or key target‐site nucleotides, red brackets mark the inserted nucleotide, and red text on the right denotes the corresponding mutation type.

**Table S1.** 3‐ to 5‐day‐old shoot tip gene editing efficiency.
**Table S2.** 7‐day‐old shoot tip gene editing efficiency.
**Table S3.** 3‐ to 5‐day‐old shoot tip gene editing efficiency of different alfalfa varieties.
**Table S4.** 3‐ to 4‐day‐old shoot tip transformation efficiency in other species.
**Table S5.** Young stem segment transformation efficiency and continuous transgene detection in transformants.
**Table S6.** Young stem segment transformation efficiency of different varieties.
**Table S7.** Young stem segment gene editing efficiency.
**Table S8.** sgRNA sequences in this study.
**Table S9.** Culture medium compositions used in this study.
**Table S10.** Primers used in this study.

## Data Availability

The data supporting the findings of this study are available within the paper and its Supplementary Information.

## References

[tpj71195-bib-0001] Brandizzi, F. , Mortimer, J. & Denby, K. (2025) Plant engineering: advances, bottlenecks, and promise. The Plant Journal, 122, e70117. Available from: 10.1111/tpj.70117 PMC12204594 40275776

[tpj71195-bib-0002] Cao, X. , Xie, H. , Song, M. , Lu, J. , Ma, P. , Huang, B. et al. (2023) Cut–dip–budding delivery system enables genetic modifications in plants without tissue culture. The Innovation, 4, 100345. Available from: 10.1016/j.xinn.2022.100345 PMC9661722 36387605

[tpj71195-bib-0003] Cao, X. , Xie, H. , Song, M. , Zhao, L. , Liu, H. , Li, G. et al. (2024) Simple method for transformation and gene editing in medicinal plants. Journal of Integrative Plant Biology, 66, 17–19. Available from: 10.1111/jipb.13593 38078380

[tpj71195-bib-0004] Cao, X. , Xie, H. , Wang, Z. , Guo, R. , Jing, F. , He, Y. et al. (2026) An efficient tissue‐culture‐free soybean genetic transformation technology using the extremely simple cut‐dip‐budding strategy. The Innovation, 7, 101221. Available from: 10.1016/j.xinn.2025.101221 PMC12957558 41789138

[tpj71195-bib-0005] Chen, C. , Hu, Y. , Ikeuchi, M. , Jiao, Y. , Prasad, K. , Su, Y.H. et al. (2024) Plant regeneration in the new era: from molecular mechanisms to biotechnology applications. Science China Life Sciences, 67, 1338–1367. Available from: 10.1007/s11427-024-2581-2 38833085

[tpj71195-bib-0006] Chen, H. , Zeng, Y. , Yang, Y. , Huang, L. , Tang, B. , Zhang, H. et al. (2020) Allele‐aware chromosome‐level genome assembly and efficient transgene‐free genome editing for the autotetraploid cultivated alfalfa. Nature Communications, 11, 2494. Available from: 10.1038/s41467-020-16338-x PMC7237683 32427850

[tpj71195-bib-0007] Chi, W. , Ma, J. , Zhang, D. , Guo, J. , Chen, F. , Lu, C. et al. (2008) The pentatricopeptide repeat protein DELAYED GREENING1 is involved in the regulation of early chloroplast development and chloroplast gene expression in *Arabidopsis* . Plant Physiology, 147, 573–584. Available from: 10.1104/pp.108.116194 PMC2409026 18400937

[tpj71195-bib-0008] Chi, W. , Mao, J. , Li, Q. , Ji, D. , Zou, M. , Lu, C. et al. (2010) Interaction of the pentatricopeptide‐repeat protein DELAYED GREENING 1 with sigma factor SIG6 in the regulation of chloroplast gene expression in *Arabidopsis* cotyledons. The Plant Journal, 64, 14–25. Available from: 10.1111/j.1365-313X.2010.04304.x 20626654

[tpj71195-bib-0009] Corona, V. , Aracri, B. , Kosturkova, G. , Bartley, G.E. , Pitto, L. , Giorgetti, L. et al. (1996) Regulation of a carotenoid biosynthesis gene promoter during plant development. The Plant Journal, 9, 505–512. Available from: 10.1046/j.1365-313X.1996.09040505.x 8624513

[tpj71195-bib-0010] Fu, C. , Hernandez, T. , Zhou, C. & Wang, Z.‐Y. (2015) Alfalfa (*Medicago sativa* L.). In: Wang, K. (Ed.) Agrobacterium protocols, Vol. 1. New York: Springer, pp. 213–221. Available from: 10.1007/978-1-4939-1695-5_17 25300843

[tpj71195-bib-0011] He, Y. , Zhang, T. , Sun, H. , Zhan, H. & Zhao, Y. (2020) A reporter for noninvasively monitoring gene expression and plant transformation. Horticulture Research, 7, 152. Available from: 10.1038/s41438-020-00390-1 PMC7502077 33024566

[tpj71195-bib-0012] Lee, H. , Chah, O.‐K. & Sheen, J. (2011) Stem‐cell‐triggered immunity through CLV3p–FLS2 signalling. Nature, 473, 376–379. Available from: 10.1038/nature09958 PMC3098311 21499263

[tpj71195-bib-0013] Li, B. , Sun, C. , Li, J. & Gao, C. (2024) Targeted genome‐modification tools and their advanced applications in crop breeding. Nature Reviews Genetics, 25, 603–622. Available from: 10.1038/s41576-024-00720-2 38658741

[tpj71195-bib-0014] Li, C. , Shen, X. , Jing, Y. , Hao, Z. , Yang, R. , Wu, J. et al. (2026) *MsSGR* interacts with *MsMSRB5* to antagonistically regulate salt tolerance in *Medicago sativa* . The Crop Journal, 14, 1242–1254. Available from: 10.1016/j.cj.2026.03.016

[tpj71195-bib-0015] Li, L. , Liu, S. , Wang, Y. , Shang, Y. , Qi, Z. , Lin, H. et al. (2024) Transcriptomic analysis of self‐incompatibility in alfalfa. Plants, 13, 875. Available from: 10.3390/plants13060875 PMC10975240 38592914

[tpj71195-bib-0016] Liu, D. , Ellison, E.E. , Myers, E.A. , Donahue, L.I. , Xuan, S. , Swanson, R. et al. (2024) Heritable gene editing in tomato through viral delivery of isopentenyl transferase and single‐guide RNAs to latent axillary meristematic cells. Proceedings of the National Academy of Sciences of the United States of America, 121, e2406486121. Available from: 10.1073/pnas.2406486121 PMC11441571 39284063

[tpj71195-bib-0017] Ogura, N. , Sasagawa, Y. , Ito, T. , Tameshige, T. , Kawai, S. , Sano, M. et al. (2023) *WUS*CHEL‐RELATED HOMEOBOX 13 suppresses de novo shoot regeneration via cell fate control of pluripotent callus. Science Advances, 9, eadg6983. Available from: 10.1126/sciadv.adg6983 PMC10328406 37418524

[tpj71195-bib-0018] Pereira, L.F.P. & Erickson, L. (1995) Stable transformation of alfalfa (*Medicago sativa* L.) by particle bombardment. Plant Cell Reports, 14, 290–293. Available from: 10.1007/BF00232030 24186762

[tpj71195-bib-0019] Samac, D.A. & Austin‐Phillips, S. (2006) Alfalfa (*Medicago sativa* L.). In: Wang, K. (Ed.) Agrobacterium protocols. Totowa, NJ: Humana Press, pp. 301–311. Available from: 10.1385/1-59745-130-4:301 16988354

[tpj71195-bib-0020] Santoro, D.F. , Bellucci, M. , De Marchis, F. , Nicolia, A. , Micheli, M. & Rosellini, D. (2025) CRISPR‐Cas9‐mediated genome editing in tetraploid alfalfa: data on efficiency, timing, and transmission to the sexual and clonal progeny. Plant Cell, Tissue and Organ Culture, 163, 111. Available from: 10.1007/s11240-025-03303-z

[tpj71195-bib-0021] Wang, Z.‐Y. , Seto, H. , Fujioka, S. , Yoshida, S. & Chory, J. (2001) BRI1 is a critical component of a plasma‐membrane receptor for plant steroids. Nature, 410, 380–383. Available from: 10.1038/35066597 11268216

[tpj71195-bib-0022] Weiss, T. , Crisp, P.A. , Rai, K.M. , Song, M. , Springer, N.M. & Zhang, F. (2022) Epigenetic features drastically impact CRISPR‐Cas9 efficacy in plants. Plant Physiology, 190, 1153–1164. Available from: 10.1093/plphys/kiac285 PMC9516779 35689624

[tpj71195-bib-0023] Xing, H.‐L. , Dong, L. , Wang, Z.‐P. , Zhang, H.‐Y. , Han, C.‐Y. , Liu, B. et al. (2014) A CRISPR/Cas9 toolkit for multiplex genome editing in plants. BMC Plant Biology, 14, 327. Available from: 10.1186/s12870-014-0327-y PMC4262988 25432517

[tpj71195-bib-0024] Yi, X. , Wang, C. , Yuan, X. , Zhang, M. , Zhang, C. , Qin, T. et al. (2024) Exploring an economic and highly efficient genetic transformation and genome‐editing system for radish through developmental regulators and a visible reporter. The Plant Journal, 120, 1682–1692. Available from: 10.1111/tpj.17068 39387436

[tpj71195-bib-0025] Youngstrom, C. , Wang, K. & Lee, K. (2025) Unlocking regeneration potential: harnessing morphogenic regulators and small peptides for enhanced plant engineering. Plant Journal, 121, e17193. Available from: 10.1111/tpj.17193 PMC11771577 39658544

[tpj71195-bib-0026] Zhai, N. & Xu, L. (2021) Pluripotency acquisition in the middle cell layer of callus is required for organ regeneration. Nature Plants, 7, 1453–1460. Available from: 10.1038/s41477-021-01015-8 34782770

[tpj71195-bib-0027] Zhang, H. , Huang, Q.‐M. & Su, J. (2010) Development of alfalfa (*Medicago sativa* L.) regeneration system and Agrobacterium‐mediated genetic transformation. Agricultural Sciences in China, 9, 170–178. Available from: 10.1016/S1671-2927(09)60081-X

[tpj71195-bib-0028] Zhao, B. & Wang, J.‐W. (2024) Perenniality: from model plants to applications in agriculture. Molecular Plant, 17, 141–157. Available from: 10.1016/j.molp.2023.12.011 38115580

[tpj71195-bib-0029] Zhao, H. , Zhao, S. , Cao, Y. , Jiang, X. , Zhao, L. , Li, Z. et al. (2024) Development of a single‐transcript CRISPR/Cas9 toolkit for efficient genome editing in autotetraploid alfalfa. The Crop Journal, 12, 788–795. Available from: 10.1016/j.cj.2024.04.001

[tpj71195-bib-0030] Zhou, A. , Wang, H. , Walker, J.C. & Li, J. (2004) BRL1, a leucine‐rich repeat receptor‐like protein kinase, is functionally redundant with BRI1 in regulating *Arabidopsis* brassinosteroid signalling. Plant Journal, 40, 399–409. Available from: 10.1111/j.1365-313X.2004.02214.x 15469497

[tpj71195-bib-0031] Zhou, C. , Han, L. , Pislariu, C. , Nakashima, J. , Fu, C. , Jiang, Q. et al. (2011) From model to crop: functional analysis of a STAY‐GREEN gene in *Medicago truncatula* and effective use of the gene for alfalfa improvement. Plant Physiology, 157, 1483–1496. Available from: 10.1104/pp.111.185140 PMC3252161 21957014

[tpj71195-bib-0032] Zhu, H. , Li, C. & Gao, C. (2020) Applications of CRISPR–Cas in agriculture and plant biotechnology. Nature Reviews Molecular Cell Biology, 21, 661–677. Available from: 10.1038/s41580-020-00288-9 32973356

