# Supplementary material for: An efficient gene delivery method utilizing the regenerative capacity of alfalfa (Medicago sativa)
Source: Plant J. 2026 Oct 29;128(3):e71195. doi: 10.1111/tpj.71195 (PMC13720224; doi:10.1111/tpj.71195)
Supplement: Supplementary file 1 [file TPJ-128-0-s001.docx]

**Supplementary Table 1. 3-5-day-old shoot tip gene editing efficiency.**

| **Varities** | **Gene** | **Explants** | **Regenerated plants** | **Edited plants** | **Homozygous** | **Heterozygous** | **Chimeric plants** |
| --- | --- | --- | --- | --- | --- | --- | --- |
| Tourist | *MsPDS* | 300 | 279(93%) | 92(33.0%) | 17(6.1%) | 75(26.9%) | 0(0%) |

**Supplementary Table 2. 7-day-old shoot tip gene editing efficiency.**

| **Varities** | **Gene** | **Explants** |  | **Regenerated plants** | **Edited plants** | **Chimeric plants** |
| --- | --- | --- | --- | --- | --- | --- |
| Tourist | *MsPDS* | 150 |  | 131(87.3%) | 17(13.0%) | 15(11.5%) |

**Supplementary Table 3.** 3-5-day-old shoot tip gene editing efficiency of different alfalfa varieties.

| **Varities** | **Gene** | **Explants** | **Regenerated plants** | **Edited plants** | **Homozygous** | **Heterozygous** | **Chimeric plants** |
| --- | --- | --- | --- | --- | --- | --- | --- |
| XJDY | *MsDG1* | 150 | 103(68.7%) ^b^ | 46(44.7%) ^a^ | 17(16.5%) | 29(28.2%) | 0(0%) |
|  | *MsSIG6* | 150 | 109(72.7%) ^b^ | 65(59.6%) ^a^ | 30(27.5%) | 35(32.1%) | 0(0%) |
|  | *MsBRI1* | 150 | 113(75.3%) ^b^ | 45(39.8%) ^a^ | 19(21.2%) | 24(21.2%) | 0(0%) |
| Pegasis | *MsDG1* | 60 | 50(83.3%) ^ab^ | 25(50.0%) ^a^ | 6(12.0%) | 19(38.0%) | 0(0%) |
|  | *MsSIG6* | 60 | 55(91.7%) ^a^ | 13(23.6%) b | 3(5.5%) | 10(18.2%) | 0(0%) |
|  | *MsBRI1* | 60 | 55(91.7%) ^a^ | 17(30.9%) ^a^ | 4(7.3%) | 13(23.6%) | 0(0%) |
| Sardi 10 | *MsDG1* | 50 | 46(92%) ^a^ | 5(10.9%) b | 0(0.0%) | 5(10.9%) | 0(0%) |
|  | *MsSIG6* | 50 | 40(80%) ^ab^ | 24(60.0%) ^a^ | 6(15.0%) | 18(45.0%) | 0(0%) |
|  | *MsBRI1* | 50 | 32(64%) ^b^ | 11(34.4%) ^a^ | 3(9.4%) | 8(25.0%) | 0(0%) |
| Paola | *MsDG1* | 50 | 20(40%) ^c^ | 10(50.0%) ^a^ | 2(10.0%) | 8(40.0%) | 0(0%) |
|  | *MsSIG6* | 50 | 37(74%) ^ab^ | 14(37.8%) ^a^**^b^** | 4(10.8%) | 10(27.0%) | 0(0%) |
|  | *MsBRI1* | 50 | 30(60%) ^b^ | 6(20.0%) ^a^ | 1(3.3%) | 5(16.7%) | 0(0%) |

**Supplementary Table 4.**3-4-day-old shoot tip transformation efficiency in other species.

| **Speices** | **Gene** | **Explants** | **Postive plants** |
| --- | --- | --- | --- |
| *S. lycopersicum* | *EGFP* | 48 | 3(6.3%) |
| *N. tabacum* | *EGFP* | 30 | 3(10.0%) |

**Supplementary Table 5.** Young stem segment transformation efficiency and continuous transgene detection in transformants.

| **Varities** | **Gene** | **Primary branch** | **Secondary branch** | **Tertiary branch** |
| --- | --- | --- | --- | --- |
| WL363 | *EGFP* | 38/38(100%) | 20/22(90.9%) | 10/14(71.4%) |
| WL363 | *Hyg* | 38/38(100%) | 22/22(100%) | 14/14(100%) |

**Supplementary Table 6.** Young stem segment transformation efficiency of different varities.

| **Varities** | **Gene** | **Explants** | **Regenerated plants** | **Postive plants** |
| --- | --- | --- | --- | --- |
| WL363 | *MsSGR* | 40 | 24(60%) | 8(33.3%) ^a^ |
| Pegasis | *MsSGR* | 40 | 36(90%) | 17(47.2%) ^a^ |
| Paola | *MsSGR* | 40 | 36(90%) | 16(44.4%) ^a^ |

**Supplementary Table 7. Young stem segment gene editing efficiency.**

| **Varities** | **Gene** | **Explants** | **Edited plants** | **Homozygous** | **Heterozygous** |
| --- | --- | --- | --- | --- | --- |
| WL363 | *MsDG1* | 60 | 16(26.7%) | 1(1.7%) | 15(25.0%) |

**Supplementary Table 8. sgRNA sequence in this study.**

| **Locus** | **sgRNA** | **Target sequence（5'-3'）** |
| --- | --- | --- |
| *MsPDS* | sgRNA | TCCTTCCATCTCCGTTAAA |
| *MsSIG6* | sgRNA1 | GCTGAATGGGCTGAGGGTGCAGG |
|  | sgRNA2 | GATTTGCTAGTTATGCATATTGG |
| *MsDG1* | sgRNA1 | GTGAGAACTTTCTGGAAGGA |
|  | sgRNA2 | GAGATGGCAAGATGCTACTCTGG |
| *MsBRI1* | sgRNA1 | CCTCTCTCCCACACCAAGTGCAC |
|  | sgRNA2 | GTTTCTGGTAACCAGTTCACCGG |

**Supplementary Table 9. Culture Medium Compositions used in this study..**

| **Medium** | **Composition** |
| --- | --- |
| Seed Germination Medium | 1/2 MS + 12 g/L agar + 10 g/L sucrose, pH 5.7 |
| Co-cultivation Medium | MS + 8 g/L agar + 30 g/L sucrose + 100 μmol/L acetosyringone + 0.4 mg/L kinetin (KT), pH 5.4 |
| MS Liquid Medium | MS + 30 g/L sucrose + 100 μmol/L acetosyringone, pH 5.4 |
| Selection Medium | MS + 8 g/L agar + 30 g/L sucrose + 1 mg/L hygromycin + 0.5 g/L timentin, pH 5.8 |
| Secondary Selection Medium | MS + 8 g/L agar + 30 g/L sucrose + 3 mg/L hygromycin + 0.5 g/L timentin, pH 5.8 |

**Supplementary Table 10 Primers** **used in this study.**

| **Primer name** | **Sequence (5'-3')** | **Experiment** |
| --- | --- | --- |
| SIG6-F | GCTGAATGGGCTGAGGGTGCAGG | Amplify *MsSIG6* |
| SIG6-R | GATTTGCTAGTTATGCATATTGG |  |
| SIG6-F1 | AGAGTCGCAGTTGGTTGTTCAG | Amplify *MsSIG6* target region |
| SIG6-R1 | CTTTGGTTGGGTTAAGATTTCC |  |
| SIG6-t1-F2 | CCCTTAAATGTATGAGATACTC | Amplify *MsSIG6* target region |
| SIG6-t1-R2 | GCAATGTAGTGTACCAAGC |  |
| SIG6-t2-F2 | GCACAGGCCTCGTAAAGAG | Amplify *MsSIG6* target region |
| SIG6-t2-R2 | GCACCGACATTTCAAAAGAG |  |
| DG1-F | GTGAGAACTTTCTGGAAGGAAGG | Amplify *MsDG1* |
| DG1-R | GAGATGGCAAGATGCTACTCTGG |  |
| DG1-F1 | AAACCTAACAGAGCAACTTATGGA | Amplify *MsDG1* target region |
| DG1-R1 | TCAGCATGGTATTTACAGTCCCTA |  |
| DG1-t1-F | GCAGAGAAGTGGTGAAGTTCC | Amplify *MsDG1* target region |
| DG1-t1-R | GTTCCCATAACGCCTCTTCTT |  |
| DG1-t2-F | CAGCCAGTGTTTATTATGAG | Amplify *MsDG1* target region |
| DG1-t2-R | CAACATTAATGGTTCCTTAGG |  |
| BRI1-F | CCTCTCTCCCACACCAAGTGCAC | Amplify *MsBRI1* |
| BRI1-R | GTTTCTGGTAACCAGTTCACCGG |  |
| BRI1-F1 | ACCTGCAACCAAACCACCATAA | Amplify *MsBRI1* target region |
| BRI1-R1 | ATCTCCGATAAAACTTCCACCT |  |
| BRI1-T1-F | ACCTGCAACCAAACCACCATAA | Amplify *MsBRI1* target region |
| BRI1-T1-R | GATGAAGAGAGTGTGTTTAGTG |  |
| EGFP-F | ATGGTGAGCAAGGGCGAGGAGCTG | Amplify *EGFP* |
| EGFP-R | TTACTTGTACAGCTCGTCCATGCCGAGAG |  |
| RUBY-F | ATGGATCATGCGACCCTCGC | Amplify *RUBY* segment |
| RUBY-R | TTGAACGTACTCGTAGAGCTG |  |
| MsActin-F | GACAATGGAACTGGAATGG | qPCR reference gene |
| MsActin-R | CAATACCGTGCTCAATGG |  |
| MsSGR-F | TCAGCCATGTCAAGAAAATTGC | qPCR target gene |
| MsSGR-R | GGCATTGGGCTCAACTTCAA |  |
| MsWUS-F | TCTGCTGGGCTTTCTTCTGC | qPCR target gene |
| MsWUS-R | GCCAATCCATCCTAAGTGAGGG |  |
| MsBBM-F | CACCTTCCGACTCATCAGGC | qPCR target gene |
| MsBBM-R | GGTGGTTGGTTTCTGAGCCA |  |
| MsPLT5-F | CAAGTACTGGGGTCCAACGAC | qPCR target gene |
| MsPLT5-R | CCATGCTGGTGGTGTCTTGT |  |
| MsWOX5-F | TGTGGTCGTTGGAATCCCAC | qPCR target gene |
| MsWOX5-R | GTGCTTGGAGTTCTGAGCCC |  |
| MsCYCB1;1-F | CTAGGTGATGCTGCCTTGGG | qPCR target gene |
| MsCYCB1;1-R | AAACTCCTTGTGACAGGGCG |  |
| EF-1α-F | ATTGGAACTGTGCCTGTGGG | qPCR reference gene |
| EF-1α-R | AGGAAGGGCCTCTGTGAGAG |  |
| Hyg-F | CCAAGCTCTGATAGAGTTGGTC | Amplify hyg fragment |
| Hyg-R | GGATTTCGGCTCCAACAATGTC |  |
